# Molecular features of heterogeneous vancomycin-intermediate *Staphylococcus aureus *strains isolated from bacteremic patients

**DOI:** 10.1186/1471-2180-9-189

**Published:** 2009-09-04

**Authors:** Yasmin Maor, Levona Lago, Amir Zlotkin, Yeshayahu Nitzan, Natasha Belausov, Debby Ben-David, Nathan Keller, Galia Rahav

**Affiliations:** 1 Infectious Diseases Unit, Sheba Medical Center, Tel Hashomer, Israel; 2 Faculty of Medicine, Tel Aviv University, Israel; 3 The Mina and Everard Goodman Faculty of Life Sciences, Bar Ilan University, Israel; 4 Microbiology Laboratory, Sheba Medical Center, Tel Hashomer, Israel

## Abstract

**Background:**

Heterogeneous vancomycin-intermediate *Staphylococcus aureus *(hVISA) bacteremia is an emerging infection. Our objective was to determine the molecular features of hVISA strains isolated from bacteremic patients and to compare them to methicillin resistant *S. aureus *(MRSA) and methicillin sensitive *S. aureus *(MSSA) blood isolates.

**Results:**

We assessed phenotypic and genomic changes of hVISA (n = 24), MRSA (n = 16) and MSSA (n = 17) isolates by PCR to determine staphylococcal chromosomal cassette (SCC*mec*) types, Panton-Valentine leukocidin (PVL) and the accessory gene regulator (*agr*) loci. Biofilm formation was quantified. Genetic relatedness was assessed by PFGE. PFGE analysis of isolates was diverse suggesting multiple sources of infection. 50% of hVISA isolates carried SCC*mec *type I, 21% type II; 25% type V; in 4% the SCC*mec *type could not be identified. Among MRSA isolates, 44% were SCC*mec *type I, 12.5% type II, 25% type V, 12.5% were non-typable, and 6% were SCC*mec *type IVd. Only one hVISA isolate and two MSSA isolates carried the PVL. Biofilm formation and *agr *patterns were diverse.

**Conclusion:**

hVISA isolates were diverse in all parameters tested. A considerable number of hVISA and MRSA strains carried the SCC*mec *type V cassette, which was not related to community acquisition.

## Background

In recent years there have been growing numbers of reports from many countries of strains of *Staphylococcus aureus *showing heterogeneous intermediate resistance to vancomycin (hVISA). The frequency of heteroresistance among MRSA isolates has recently reached 6% to 11% [1-3]. In our institution there are approximately 200 *S. aureus *bacteremias each year. Of these, 50% are MRSA and 6% demonstrate hVISA resistance [2,3]. Molecular assessment of the clonal dissemination of hVISA isolates has yielded conflicting results. Several studies found genetic linkage between hVISA isolates, reflected by a single pulsed field gel electrophoresis (PFGE) clone [4-6], while others showed that hVISA isolates were genetically diverse [7,8].

The mechanism by which hVISA occurs is still under investigation. The hVISA phenotype has a thickened cell wall, altered peptidoglycan cross-linking, altered penicillin-binding protein expression, and slower growth rate [1-3,7]. Several genes related to cell regulation pathways have been proposed as involved in the development of resistance to glycopeptides. For example *vraSR*, *graSR saeSR*, and *agr*, [9-12], but the global mechanism of resistance and the interactions between these various pathways are not clear. Most of hVISA isolates were acquired in hospital settings, and most patients had recurrent hospitalizations, substantial comorbidities [1-3,7] and poor response to vancomycin therapy [7,8].

The staphylococcal cassette chromosome (SCC*mec*) encodes methicillin resistance as well as genes responsible for resistance to other antibiotics. At least five different types of SCC*mec *were found in *S. aureus *(SCC*mec *types I to V), and SCC*mec *types IV and V were associated with community acquired MRSA [13,14]. SCC*mec *typing has rarely been performed on hVISA isolates, and when performed, most isolates carried the SCC*mec *type I and II, similar to hospital-acquired MRSA [6,14,15].

The accessory gene regulator (*agr*) operon in *S. aureus *coordinates quorum sensing as well as virulence pathways. In general, *agr *activates genes encoding tissue-degrading factors (secreted virulence factors) and represses genes that encode factors important for colonization (virulence factors expressed on the staphylococcal cell surface). DNA sequence polymorphisms at this locus comprise four *S. aureus agr *groups (I-IV), and *S. aureus *strains of specific *agr *groups have been associated with certain clinical characteristics. In several studies performed in Japan and the USA, VISA and hVISA clinical isolates belonged to *agr *groups I or II [16,17].

Similarly, the expression of Panton-Valentine leukocidin (PVL), a two-component pore-forming cytolytic toxin that targets mononuclear and polymorphonuclear cells and causes cell death, has been strongly associated with community acquired MRSA. However, its association with hVISA strains has not been defined yet [18].

Many staphylococcal infections are associated with communicating cell groups known as biofilms in which cells are attached to abiotic or biotic surfaces and posses altered growth rates and altered gene expression profiles compared to planktonic bacteria. It was demonstrated that hVISA isolates that belonged to *agr*-group II were defective in *agr*-function; conversely, these strains were strong biofilm producers. These findings led to the hypothesis that VISA strains may exhibit diminished virulence and might have an enhanced ability to form a thick biofilm due to *agr*-locus inactivation [16].

The purpose of this study was to assess the clonal dynamics of hVISA bacteremia in our hospital, to carry out comprehensive phenotypic and genotypic analyses of hVISA, MRSA and MSSA blood isolates recovered in Israel, and to determine whether any additional phenotypic or genotypic characteristic could be used in the recognition of hVISA.

## Results

The study included 24 hVISA isolates, 16 MRSA isolates and 17 MSSA isolates. All hVISA isolates were identified as such by the Etest macromethod and the hVISA phenotype was confirmed by population analysis in all cases. All MRSA and MSSA isolates did not demonstrate heteroresistance to vancomycin as shown by the etest macromethod.

### PFGE of hVISA isolates

**Figure 1:**
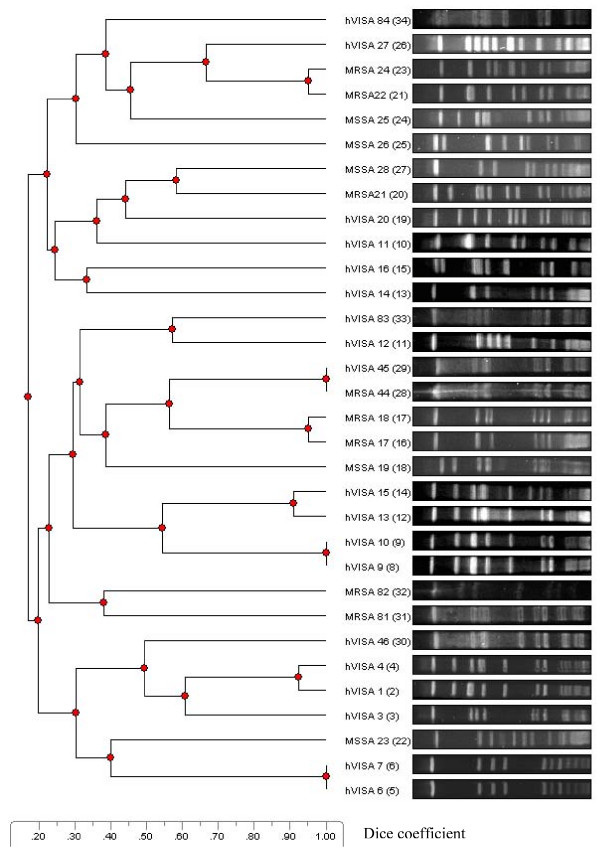
**PFGE of hVISA, MRSA and MSSA isolates**.

The PFGE profiles of hVISA isolates exhibited a large diversity. Of the 18 isolates examined, 15 different pulsotypes were found, suggesting concomitant multiple sources of infection (Figure 1). In two cases similar hVISA pulsotypes between two patients were identified. Similarly, there was a great diversity in the pulsotypes of the MRSA isolates tested; only one of the MRSA pulsotypes was similar to one of the hVISA pulsotypes.

### SCCmec type

**Figure 2:**
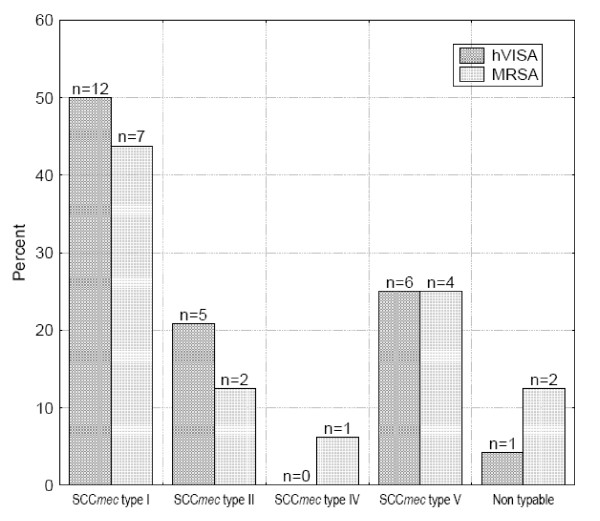
***SCC*mec typing among hVISA and MRSA isolates using Zhang's method **[32].

Fifty percent (n = 12), 21% (n = 5) and 25% (n = 6) of the hVISA isolates carried SCC*mec *type I, *SCCmec *type II and *SCCmec *type V, respectively. Ten isolates that were nontypable using Olivera's method carried *SCCmec *type V by Zhang's method, except one isolate that was nontypable by both methods (Figure 2). The distribution of *SCCmec *types among the16 MRSA isolates revealed SCC***mec ***type I in 44% (n = 7), type V in 25% (n = 4), type II in 12.5% (n = 2) and type IVd in 6% (n = 1). Two isolates were nontypable using both methods. None of the hVISA or MRSA isolates with *SCCmec *type IV or V had antibiotic susceptibility patterns compatible with community acquisition (Table 1), as almost all isolates were resistant to gentamicin and fluoroquinolones. However, the majority of these isolates were susceptible to erythromycin and clindamycin.

**Table 1:** Antibiotic susceptibility of hVISA and MRSA isolates which carried SCC*mec *type IV and V

	SCC*mec *type	RIF	CLIN	ERYT	GENT	AMIK	OFL	FUS	TMP-SMZ
**hVISA** **Isolate #**									
25717	V	S	R	R	R	S	R	S	S
24406	V	S	S	S	R	S	R	S	S
18981	V	R	S	S	R	S	R	S	S
34139	V	S	S	S	R	S	R	S	S
22035	V	S	S	S	R	S	R	S	S
7555	V	S	S	S	R	S	R	S	S

**MRSA** **Isolate #**									
13372	V	S	R	R	R	S	R	S	S
13468	V	S	R	R	S	S	R	S	S
41901	V	S	S	S	R	S	R	S	S
41168	V	S	S	S	R	S	R	S	S
13731	IVd	S	S	S	R	S	R	R	R

Antibiotic susceptibility of isolates was tested using the disc diffusion method according to the CLSI guidelines.

### PVL genes

Only one hVISA isolate and two MSSA isolates carried PVL. Furthermore, even the MRSA isolate with SCCmec type IVd did not carry the PVL gene.

### Agr-genotype

**Figure 3:**
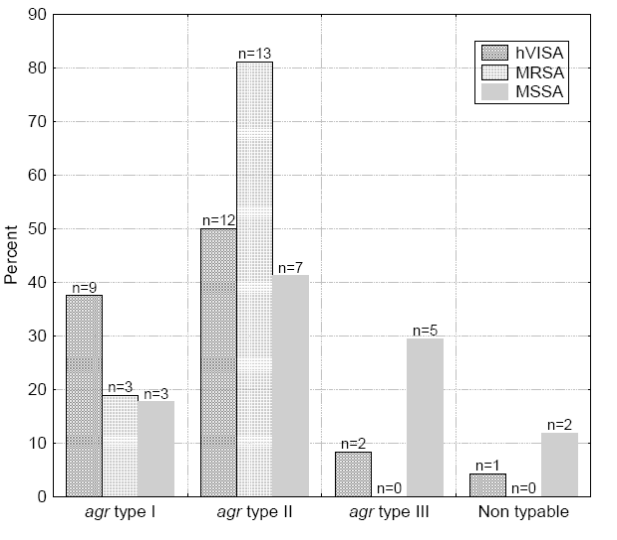
***agr *typing among hVISA, MRSA and MSSA isolates**.

All agr types were represented in the 24 isolates of hVISA (Figure 3): 37.5% were agr-group I, 50.0% agr-group II, 8.4% agr-group III and 4.1% were non-typable. The 16 isolates of MRSA carried agr-group I (18.8%) and agr-group II (81.2%). The 17 isolates of MSSA carried agr-group I (17.6%), agr-group II (41.2%) or agr group III (29.4%), and 11.8% were non-typable.

### Biofilm

Determination of biofilm production Quantitative determination of biofilm formation showed a strong biofilm production in 6 of 24 isolates (25%) of hVISA, 9 of 16 isolates of MRSA (55.5%) and 5 of 17 MSSA isolates (29%). There was no relation between biofilm production and agr group.

## Discussion

Molecular assessment of hVISA isolates indicated a number of PFGE groups, with no substantive evidence of clonal dissemination. Isolates that appeared to be clonal were generally not epidemiologically linked by department or by time. Although the molecular epidemiology of the MRSA isolates in hospitals in Israel has not been explored yet, the high diversity among MRSA isolates in our study is remarkable. In previous reports, VISA and hVISA strains described in Europe belonged to a restricted range of epidemic multidrug-resistant MRSA strains [4-8], a worrisome finding that highlighted the potential of MRSA strains with reduced susceptibility to vancomycin to become widespread. However, in our study, genetic lineage was not demonstrated between the hVISA and MRSA isolates. All hVISA isolates had a similar resistance profile to multiple antimicrobial agents, including aminoglycosides and fluoroquinolones. This association between hVISA and a multiresistance phenotype was reported previously [19].

The majority of hVISA and MRSA isolates in the current study harbored SCC*mec *type I or II, consistent with nosocomial acquisition. However, 25% and 31% of hVISA and MRSA isolates, respectively, carried the *SCC*mec types IV or V that are related to community acquisition [13,14]; none of these patients acquired the infection in a community setting, and the antibiotic susceptibility of isolates was compatible with nosocomial acquisition. Furthermore, the PVL gene was found in only one hVISA isolate. Our study reasserted that hVISA, as well as nosocomial acquired MRSA, may carry the so-called community acquired SCC*mec *types IV and V. It is possible that these clones originated in the community and were introduced by patients who were hospitalized. However, during the study period there were no truly community-acquired cases, suggesting that community acquisition is rare.

In that respect, once introduced into the hospital, the SCC*mec *type V strains may present a competitive advantage over the predominant endemic multiresistant MRSA clones, in a similar manner SCC*mec *type IV now seen in the United States, where the multiplication and transmission rates appear superior to those of MRSA strains with other SCC*mec *types [20]. Another possibility is that *S. aureus *SCC*mec *type V is originally nosocomial and has spread to the community. In several other reports, the SCC*mec *types common among hVISA isolates were I and II [6,14,15].

Only 5.2% of the *S. aureus *isolates in this investigation contained the PVL gene, supporting the findings of another study that the prevalence of community MRSA and carriage of the PVL gene among *S. aureus *isolates in Israel is low [21]. The low prevalence of the PVL gene in our isolates may be due to the impact of geography on the genetic make-up of *S. aureus*. Strains of MSSA causing skin and soft tissue infections in South Africa were significantly more likely to contain a variety of toxins or leukocidins, including PVL, than MSSA isolates causing similar infections from the United States [22]. The current study did not focus on *S. aureus *isolated from skin and soft tissue infections, a clinical condition with which PVL has been strongly associated, and this might also explain the above observations.

In several studies on *agr *groups among VISA/hVISA strains, most isolates had *agr *II polymorphism. It was suggested that loss of function of the *agr *operon might confer a survival advantage to *S. aureus *under vancomycin selection pressure, particularly in strains with the *agr *group II genotype [16,17]. In the present study, *agr *II was the most common *agr *group among MRSA isolates; hVISA isolates on the other hand, demonstrated high diversity in *agr *polymorphism, which supports the suggestion that *agr *is probably not associated with the development of resistance to vancomycin.

Reports regarding biofilm formation and hVISA are conflicting. Some demonstrated a reduction of biofilm formation among hVISA isolates [23], while others documented an increase [24]. Although hVISA infections are associated with the presence of foreign bodies [7], we could not find high incidence of biofilm producers among the hVISA isolates.

## Conclusion

hVISA isolates are genetically diverse in their PFGE profile, their SSC*mec *and *agr *types, and most strains in Israel do not harbor the PVL genes. A considerable number of hVISA and MRSA isolates in Israel carried SCC*mec *type V cassette, which was not related to community acquisition.

## Methods

All blood isolates of hVISA that were identified during 2003 to 2006 at the Sheba Medical Center, a tertiary care center with 1,480 beds, affiliated ambulatory clinics and long-term care facilities, were included (n = 24). Sixteen and 17 randomly selected blood isolates of MRSA and methicillin sensitive S. aureus (MSSA), respectively, formed the control groups. The study was approved by Sheba's Institutional Review Board.

### Identification and confirmation of methicillin and intermediate vancomycin resistance

During 2003-2004, resistance to methicillin was identified by the Kirbi-Bauer oxacillin disk diffusion method. Thereafter the method was changed to the cefoxitin disk diffusion method detailed by the Clinical and Laboratory Standards Institute [25,26]. All isolates included in the study were assessed for the presence of hVISA by the Etest macromethod [27]. Antibiotic susceptibility tests were performed on fresh samples, because reversion of resistance after laboratory manipulation had been reported [28]. In brief, strains were grown for 18-24 hours on blood agar plates. Randomly selected single colonies were inoculated into fresh brain-heart infusion (BHI) broth. One hundred microliters of 2.0 McFarland suspensions were drawn onto BHI agar plates. Etest strips (AB Biodisk, Solna, Sweden) for vancomycin and teicoplanin were applied on the same plate, which was subsequently incubated at 35°C for 48 h. Strains were considered hVISA if readings were ≥8 μg/ml for vancomycin and teicoplanin or ≥12 μg/ml for teicoplanin alone. All isolates that were positive for hVISA using the macromethod were further tested using population analysis method as previously described [29]. Briefly, after 24 hours of incubation cultures were diluted in saline to 10^-3^, 10^-6 ^and 10^-8 ^and plated on to BHIA plates containing 0.5, 1, 2, and 4 mg/L vancomycin. Colonies were counted after 48 hours of incubation at 37°C and the viable count was plotted against vancomycin concentration. The area under the curve (AUC) was used to distinguish hVISA from glycopeptide susceptible isolates. A ration of the AUC of the test isolate was divided by the corresponding AUC for a strain validated against a Mu 3 strain (courtesy of Roland Jones, JMI Laboratories, North Liberty, IA 52317, USA). The criteria used for detection of hVISA were AUC ≥ 0.9.

### Pulsed field gel electrophoresis

Genetic relatedness of hVISA strains digested with SmaI was assessed by PFGE, as described elsewhere [30]. Strains were considered indistinguishable if there was no difference in bands, and related (i.e. variants of the same PFGE subtype) if they varied by 1 to 3 bands. A PFGE dendogram was constructed using GelCompar II (Applied Maths, Sint-Martens-Latem, Belgium) to calculate similarity coefficients and to perform unweighted pair group analysis using arithmetic mean clustering. Dice coefficient with 0.5% optimization and 1.0% position tolerance was used.

### Polymerase chain reaction (PCR) for genotyping

Genomic DNA was extracted using Wizard Genomic DNA Purification Kit (Promega, Madison, WI, USA) according to the manufacturer's protocol for Gram positive bacteria. DNA samples were stored at -20°C until used for analysis. Bacterial determinants that were examined using PCR assays included PVL, agr groups I to IV, and SCCmec types. Primers and conditions used to amplify the genes of interest are described in Table 2. Positive and negative controls were included in each PCR run.

**Table 2:** List of primers used for the various PCR reactions

Locus/Type	Primer	Nucleotide Sequence	Size (bp)
**SCC*mec***			
A	CIF2 F2	TTCGAGTTGCTGATGAAGAAGG	495
	CIF2 R2	ATTTACCACAAGGACTACCAGC	
B	KDP F1	AATCATCTGCCATTGGTGATGC	284
	KDP R1	CGAATGAAGTGAAAGAAAGTGG	
C	MECI P2	ATCAAGACTTGCATTCAGGC	209
	MECI P3	GCGGTTTCAATTCACTTGTC	
D	DCS F2	CATCCTATGATAGCTTGGTC	342
	DCS R1	CTAAATCATAGCCATGACCG	
E	RIF4 F3	GTGATTGTTCGAGATATGTGG	243
	RIF4 R9	CGCTTTATCTGTATCTATCGC	
F	RIF5 F10	TTCTTAAGTACACGCTGAATCG	414
	RIF5 R13	GTCACAGTAATTCCATCAATGC	
G	IS431 P4	CAGGTCTCTTCAGATCTACG	381
	pUB110 R1	GAGCCATAAACACCAATAGCC	
H	IS431 P4	CAGGTCTCTTCAGATCTACG	303
	pT181 R1	GAAGAATGGGGAAAGCTTCAC	
mecA	MECA P4	TCCAGATTACAACTTCACCAGG	162
	MECA P7	CCACTTCATATCTTGTAACG	

**SCC*mec *type V**			
Type I	Type I-F	GCTTTAAAGAGTGTCGTTACAGG	613
	Type I-R	GTTCTCTCATAGTATGACGTCC	
Type II	Type II-F	CGTTGAAGATGATGAAGCG	398
	Type II-R	CGAAATCAATGGTTAATGGACC	
Type III	Type III-F	CCATATTGTGTACGATGCG	280
	Type III-R	CCTTAGTTGTCGTAACAGATCG	
Type IVa	Type IVa-F	GCCTTATTCGAAGAAACCG	776
	Type IVa-R	CTACTCTTCTGAAAAGCGTCG	
Type IVb	Type IVb-F	TCTGGAATTACTTCAGCTGC	493
	Type IVb-R	AAACAATATTGCTCTCCCTC	
Type IVc	Type IVc-F	ACAATATTTGTATTATCGGAGAGC	200
	Type IVc-R	TTGGTATGAGGTATTGCTGG	
Type IVd	Type IVd-F5	CTCAAAATACGGACCCCAATACA	881
	Type IVd-R6	TGCTCCAGTAATTGCTAAAG	
Type V	Type V-F	GAACATTGTTACTTAAATGAGCG	325
	Type V-R	TGAAAGTTGTACCCTTGACACC	
mecA	MecA147-F	GTG AAG ATA TACCAAGTG ATT	147
	MecA147-R	ATG CGCTATAGATTG AAAGGAT	

**Panton-Valentine leukocidin (PVL)**			
	luk-PV-1	ATCATTAGGTAAAATGTCTGGACATGATCCA	433
	luk-PV-2	GCATCAASTGTATTGGATAGCAAAAGC	

**Accessory gene regulator (*agr*)**			
	agrSa agr1-4Sa-1	ATGCACATGG TGCACATGC	
	agr-1Sa agr1Sa-2	GTCACAAGTA CTATAAGCTG CGAT	439
	agr-2Sa agr2Sa-2	TATTACTAAT TGAAAAGTGC CATAGC	572
	agr-3Sa agr3Sa-2	GTAATGTAAT AGCTTGTATA ATAATACCCAG	321
	agr-4Sa agr4Sa-2	CGATAATGCC GTAATACCCG	657

**Gyrase**			
	gyrA-F	AGTACATCGT CGTATACTAT ATGG	280
	gyrA-R	ATCACGTAAC AGTTCAAGTGTG	

### SCCmec typing

Multiplex PCR was used to determine SCCmec type I-V on all hVISA and MRSA isolates, according to the methods published by Oliveira [31] and Zhang [32] using respectively the ReddyMix PCR master mix (ABgene, UK) and Phusion HF master Mix (Finnzymes, Finland).

### Panton-Valentine leukocidin

PVL genes were detected by co-amplification of the lukS-PV and lukF-PV genes as described by Lina [33], using the Phusion HF master Mix. S. aureus ATCC 25923 was a positive control.

### Accessory gene regulator

The agr locus was defined by multiplex PCR according to the published protocol [34].

### Assessment of biofilm formation

Biofilm formation was quantified using a colorimetric microtiter plate assay [35]. Two hundred μL of bacterial suspension were placed into the wells of sterile 96-well polystyrene U-bottom microtiter plates. After overnight incubation, media and non-adherent planktonic bacteria were removed by gentle washing of the plate with sterile normal saline. The attached bacteria were fixed by adding 99% methanol to each well, and then the wells were emptied and dried before 200 μL of 2% gentian violet 4% in 12% ethanol was added. The dye bound to the adherent cells was resolubilized by adding 200 μL of gentian violet 4% in 12% ethanol to each well. The optical density (OD) of each well was determined photometrically at 595 nm. Wells originally containing sterile medium and non-biofilm producing bacteria *Staphylococcus epidermidis*, ATCC 12228 served as a control. The test was carried out in quadruplicate. The reference value for calculating adherence was OD 0.126. This number was calculated from the blank readings as mean + 3 × SD. Readings ≤ 0.126 OD were classified as a non biofilm producer and readings > 0.126 OD as a biofilm producer [35].

### Statistical analysis

Fisher exact test was used for comparing hVISA, MRSA and MSSA results. Significance level was set at p < 0.05.

## Abbreviations

hVISA: heterogeneous vancomycin-intermediate *S. aureus; *MRSA: methicillin resistant *S. aureus*; MSSA: methicillin sensitive *S. aureus*; PCR: polymerase chain reaction; SCC*mec: *staphylococcal chromosomal cassette; PVL: Panton-Valentine leukocidin; *Agr: *accessory gene regulator; PFGE: pulsed field gel electrophoresis; AUC: area under the curve; RIF: rifampin; CLIND: clindamycin; ERYT: erythromycin; GENT: gentamicin; AMIK: amikacin; OFL: ofloxacin; FUS: fusidic acid; TMP-SMZ: trimethoprim/sulfamethoxazole; S: Sensitive; R: Resistant;

## Authors' contributions

YM conceived the study, participated in its design, performed the analysis and interpretation of the data and wrote the manuscript. LL carried out the molecular genetic studies, and participated in the interpretation of the data and writing the manuscript. AZ developed and carried out the assays assessing biofilm formation, and participated in interpreting the molecular data. YN participated in conceiving the study, its design interpretation and writing the drafted manuscript. NB identified the hVISA strains and participated in the design and interpretation of the data. DB participated in the study design, participated in analysis and interpretation of the data and in writing the manuscript. NK participated in conceiving the study design, participated in analysis and interpretation of the data and in writing the manuscript. GR participated in conceiving the study, participated in its design, participated in analysis and interpretation of the data and in writing the manuscript.
